# Spider Web-Like Phononic Crystals for Piezoelectric MEMS Resonators to Reduce Acoustic Energy Dissipation

**DOI:** 10.3390/mi10090626

**Published:** 2019-09-19

**Authors:** Fei-Hong Bao, Xue-Qian Wu, Xin Zhou, Qi-Die Wu, Xiao-Sheng Zhang, Jing-Fu Bao

**Affiliations:** 1 School of Electronic Science and Engineering, University of Electronic Science and Technology of China, Chengdu 61173, China; 2 Yingcai Honors College, University of Electronic Science and Technology of China, Chengdu 61173, China

**Keywords:** phononic crystals, piezoelectric, MEMS resonator, energy dissipation, finite-element-analysis, bandgap, transmission property, quality factor

## Abstract

Phononic crystals (PnC) are a remarkable example of acoustic metamaterials with superior wave attenuation mechanisms for piezoelectric micro-electro-mechanical systems (MEMS) resonators to reduce the energy dissipation. Herein, a spider web-like PnC (*SW-PnC*) is proposed to sufficiently isolate the wave vibration. Finite-element analysis is performed to gain insight into the transmission property of finite PnC, and band characteristics by infinite periods. In comparison with the circle hole PnC at a similar bandgap, due to its already very lightweight PnC structure compared with previously reported PnCs, the proposed PnC offers a significantly lighter weight, smaller lattice constant, and greater energy leakage inhibition. More specifically, the resonator with the *SW-PnC* plate as the anchoring substrate exhibited a quality factor as high as 66569.7 at 75.82 MHz.

## 1. Introduction

In the past decades, acoustic metamaterial has aroused a tremendous interest in lots of potential applications, including acoustic super-lensing/hyper-imaging, beam steering, and cloaking [1,2,3]. In particular, phononic crystal (PnC) is a remarkable example of acoustic metamaterials due to its salient trait, such as vibration attenuation/isolation, wave filter, localization, and acoustic wave guide [4,5,6]. For this reason, PnC has been rapidly developed, while it is fast growing in physics and engineering communities today. Besides, micro-electro-mechanical systems (MEMS) technologies have shown promising prospects in wireless communication systems and sensor networks [7,8]. More specifically, silicon-based MEMS resonators offer attractive features in complementary metal oxide semiconductor (CMOS) process compatibility, as well as high power handling capacity [9]. Furthermore, aluminum nitride (AlN) piezoelectric thin film exhibits a high phase velocity, weak phase velocity dispersion, and moderate electromechanical coupling coefficient (*k*^2^_eff_) [10]. Consequently, the AlN-on-Silicon MEMS resonator is a satisfactory technology for single-chip multi-band wireless communications [11].

In the MEMS resonator-based applications, a high-quality factor (*Q*) is highly desirable for achieving low phase noise and high frequency stability as oscillators, and realizing low insertion loss as filters [12]. Up to date, various energy loss reduction strategies for piezoelectric MEMS resonators to improve *Q* have been proposed and aroused much attention [13,14,15,16,17,18]. Moreover, exploiting one-dimensional (1D) or two-dimensional (2D) PnC in tethers or surrounding the resonant body of resonators is an effective technology to significantly reduce the anchor loss [19,20,21,22,23,24,25]. Even so, one of the critical challenges of PnC applied in resonators still faced by researchers is the large lattice constant compared to the associated acoustic wavelength. For instance, the resonant frequency of the fundamental width extensional (WE) lamb mode is decided by the half-wavelength width of the resonant body. Meanwhile, Bragg scattering acoustic bandgaps occur at wavelengths of the order of the PnC unit cell size in general [26], thus it is still difficult to exploit small-sized PnC arrays in a highly integrated MEMS device to greatly improve its performance. Given this, it is highly urgent to develop a PnC structure, with the following features of a small lattice constant, lightweight, and wide acoustic bandgaps, to enable the miniaturization of MEMS devices.

In addition, the structural behavior of orb spider webs, which is inspired by natural materials, has been studied under quasi-static and dynamic loading conditions [27,28,29]. Another silk-based spider-web structure designed as a PnC structure was firstly proposed, and its propagation characteristics of elastic waves were numerically studied [30]. However, it was found that the silk-based spider web-like PnC (*SW-PnC*) structure with a square outframe in [30] could not form any complete acoustic bandgaps in the MHz frequency range. Meanwhile, the *SW-PnC* structure has yet to be utilized for anchor loss reduction in silicon-based MEMS resonators. Thereby, we optimized the *SW-PnC* structure to successfully form three complete bandgaps in the MHz frequency range, and then applied it in MEMS resonators to reduce the acoustic energy dissipation.

In this research, a PnC unit cell with a large circle hole (*C-PnC*) was selected, because of its already very lightweight PnC structure compared with previously reported PnCs (i.e., ring-shaped PnC, cross-shaped PnC, fractal-like PnC, etc.) [19,31], to compare the lattice constant (a), weight, and energy loss suppression in a similar bandgap frequency range with *SW-PnC*. Subsequently, multiphysics finite-element-analysis (FEA) simulations of the delay line and solid line with finite PnC periods, and with the reference solid silicon slab as the transmission mediums between interdigital transducers (IDTs) were performed, verifying the existence of acoustic bandgaps that can be formed by the associated PnC structure with infinite periods and varying abilities of acoustic wave isolation for different PnCs. Finally, three types of MEMS resonators, including the conventional resonator (C), the resonator with *C-PnC* array plates (RCP), and that with *SW-PnC* array plates (RSWP) were designed to further verify the effectiveness of finite PnC structure in reducing the anchor loss and indicate that the resonator RSWP could realize the optimal *Q*.

## 2. Phononic Crystals Design

**Figure 1:**
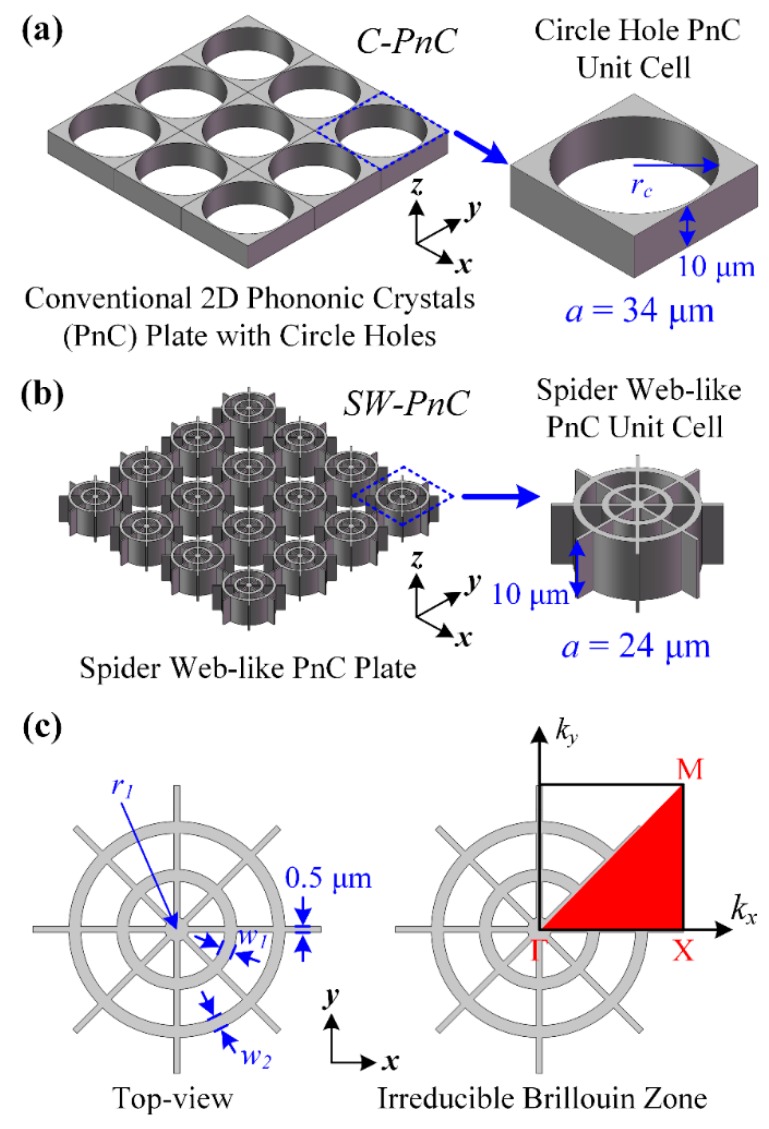
(**a**) Three-dimensional (3D) illustration of the 2D phononic crystals (PnC) plate with circle holes, and its unit cell with the definition of geometry parameters. The thickness of PnC structures was fixed as 10 μm in this research. The radius (*r*_c_) and lattice constant (*a*) of the circle hole PnC (*C-PnC*) are 16.5 and 34 μm, respectively. (**b**) Illustration of the spider web-like PnC (*SW-PnC*) plate and its unit cell. (**c**) Schematic top-view of the geometry parameters and irreducible brillouin zone for the *SW-PnC* unit cell. The lattice constant, radius (*r*_1_) of circle, and width of rings (*w*_1_, *w*_2_) of *SW-PnC* are 24, 1, 2, and 2 μm, respectively. Therefore, the geometry designs ensured the minimum width of the *SW-PnC* unit cell and *C-PnC* unit cell was the same as 0.5 μm.

Figure 1 shows the 3D models of two types of PnC plate containing a 2D PnC plate with circle holes and a 2D spider web-like PnC plate. In order to utilize these PnC structures in MEMS resonators in this research, the PnCs were made of the most commonly used anisotropic single crystalline silicon [32] and the thickness of PnC structures was fixed as 10 μm. As shown in Figure 1, the default *x* axis, *y* axis, and *z* axis was set as the (110), (–110), and (001) direction of a standard (100) direction silicon wafer. Moreover, the elasticity values of the single crystalline silicon in this research were defined as:

(1) $$\begin{array}{l} E_{x}=E_{y}=169\;\mathrm{GPa}\;\;E_{z}=130\;\mathrm{GPa}\\ \sigma_{yz}=0.36\;\;\sigma_{zx}=0.28\;\;\sigma_{xy}=0.064\\ G_{yz}=G_{zx}=79.6\;\mathrm{GPa}\;\;G_{xy}=50.9\;\mathrm{GPa} \end{array}$$

where the *E*, *σ*, and *G* are the Young’s modulus, Poisson’s ratio, and shear modulus of the adopted single crystalline silicon, respectively. Besides, the values of mass density (*ρ*) and acoustic velocity (*v*) of silicon are 2330 kg/m^3^ and 8500 m/s, respectively.

To simultaneously obtain a similar frequency range and width of the acoustic bandgap formed by the proposed PnCs, the geometry parameters of the radius (*r*_c_) and lattice constant (*a*) of the circle hole PnC (*C-PnC*) unit cell were designed as 16.5 and 34 μm, respectively. Moreover, the radius (*r*_1_) of the circle, width of the narrow beams, and width of the rings (*w*_1_, *w*_2_) of the spider web-like PnC (*SW-PnC*) unit cell were defined as 24, 1, 0.5, 2, and 2 μm, respectively. In general, in comparison with the 3 × 3 *C-PnC* array (102 μm × 102 μm), the proposed 4 × 4 *SW-PnC* array (96 μm × 96 μm) has an approximate size but offers a lighter weight and greater ability of acoustic wave isolation (i.e., which will be demonstrated later). In addition, the proposed *SW-PnC* works around the frequency range of 76 MHz. For other frequency ranges, the in-plane dimension size of the lattice constant and radii of the circles for the PnC unit cell can be reduced or enlarged proportionally. Changing the width of the beams is not recommended, as this width would be likely set at the minimum width imposed by the fabrication process. Besides, the *SW-PnC* is difficult to fabricate due to its large thicknesses/aspect ratios in the current Bosch process. So, the *SW-PnC* is at present suggested to enlarge the in-plane size to reduce the thicknesses/aspect ratios, and then use it in some lower frequency applications.

In this research, the propagation characteristics of acoustic waves of PnCs were systematically investigated by using the COMSOL (5.4, COMSOL, Inc., Burlington, MA, USA) multiphysics finite-element analysis. Specifically, wave dispersion relations in an infinite PnC lattice were studied by applying Floquet periodic boundary conditions at the unit cell boundaries, while performing a parametric sweep in the wave vector, *k*, along the borders of Γ-X-M-Γ for the first irreducible brillouin zone as depicted in Figure 1c. Furthermore, to verify that acoustic bandgaps can be formed by the associated PnC structure, transmission parameters (i.e., S21) were introduced in the PnC-based delay line and solid line to quantify the acoustic wave isolation, and the S21 in decibels was expressed as:

(2) $$S21(\mathrm{dB})=10\log_{10}(\frac{P_{\mathrm{out}}}{P_{\mathrm{in}}}),$$

where *P*_out_ and *P*_in_ are the values of the output and input power in the delay line and solid line, respectively. The initial *P*_in_ was 0 dBm. Moreover, S21 is the S-parameter of transmitted waves, and represents the power transmission coefficient from the input port to the output port. Consequently, the proposed multiphysics finite-element analysis (FEA) simulation models of the delay line and solid line can be used to map the mechanical quantities to electrical ones, and further prove the transmission properties of PnCs.

**Figure 2:**
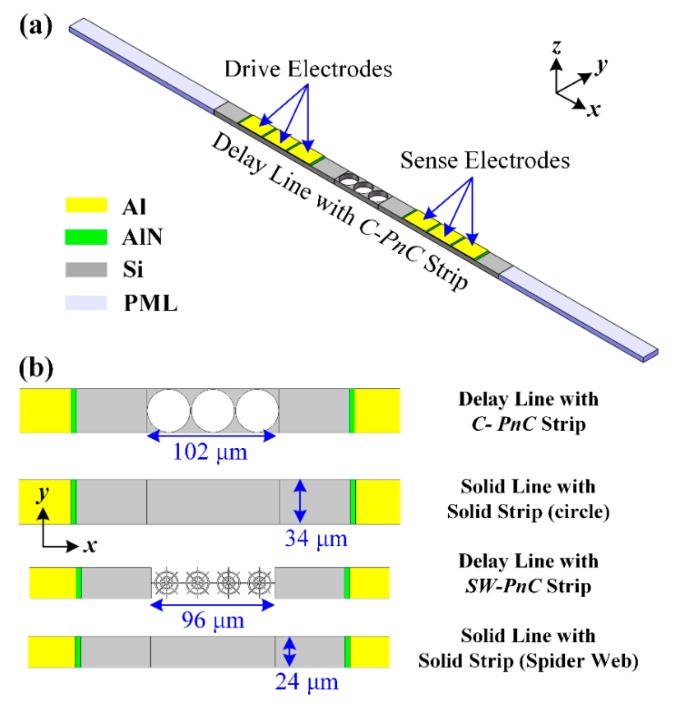
(**a**) Schematic set-up for the computation of the transmission property of the acoustic delay line with a finite C-PnC strip. Furthermore, (**b**) the delay line and solid line with the PnC strip, and with the solid silicon strip as the transmission medium between interdigital transducers (IDTs) were performed, verifying the existence of acoustic bandgaps formed by the associated PnC. In this research, periodic boundary conditions were applied to surfaces along the *y*-direction of the delay line and solid line to form the 2D PnC slab and reference solid slab, while improving the calculation efficiency.

As shown in Figure 2a, a delay line has finite PnC periods as the transmission medium between the drive and sense interdigitated transducers (IDTs), and the piezoelectric thin film is (AlN, 0.5 μm thick) sandwiched between the IDTs (Al, 1 μm thick) and the silicon substrate (single crystalline silicon, 10 μm thick). Besides, the solid line was designed by the PnC strip in the delay line, which replaces the solid slab. To avoid unexpected spurious peaks being stimulated in the transmission spectra by reflected waves, perfectly matched layers (PMLs) were introduced at the ends of the delay line and solid line. As shown in Figure 2, the width (*y*-direction) of the delay line and solid line was equal to the lattice constant (*a*) of the associated PnC while the length of PMLs (*x*-direction) was set as the 3-fold wavelength (3*λ*). In the meantime, in order to form a 2D PnC slab and improve the calculation efficiency, periodic boundary conditions were applied to surfaces along the *y*-direction of the delay line and solid line.

**Figure 3:**
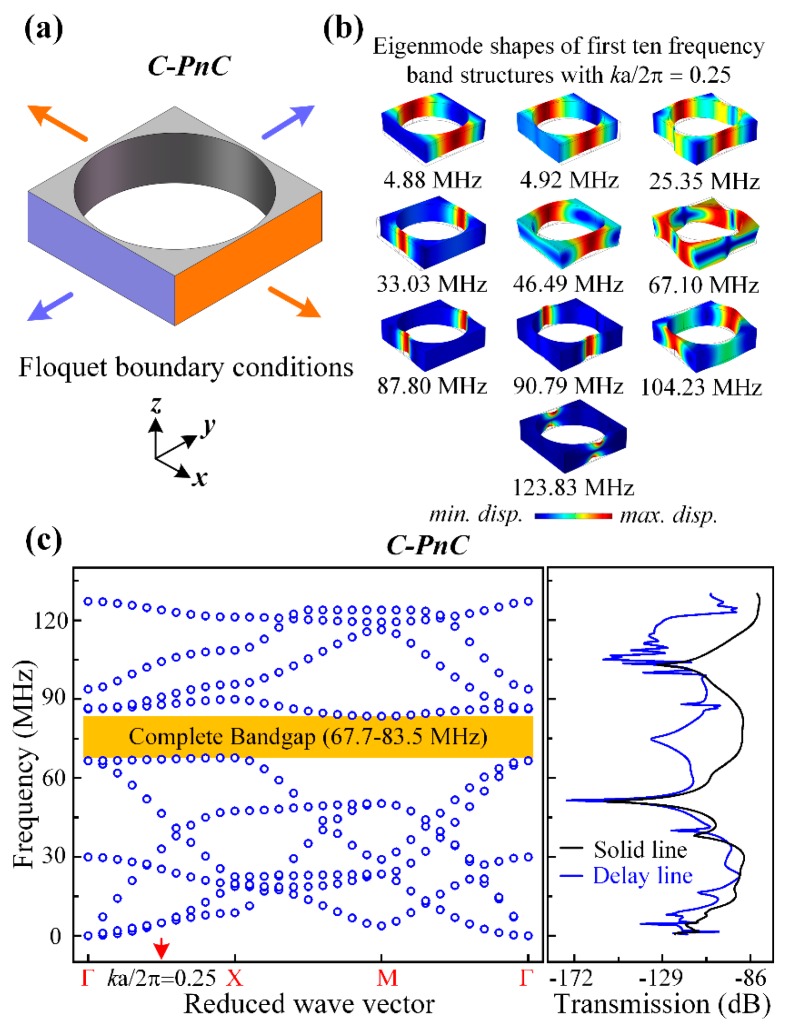
(**a**) 3D Illustration of the Floquet boundary conditions applied to a *C-PnC* unit cell. The boundary condition applied on the orange surfaces means an infinite number of repetitions in the *x*-direction, and the condition applied on the purple surfaces means an infinite number of repetitions in the *y*-direction. (**b**) Corresponding eigenmode shapes of the first 10 frequency band structures for the proposed *C-PnC*, with *k*a/2π = 0.25. (**c**) Band structures of an infinite PnC are consistent with the transmission characteristics through a finite number of PnC unit cells for *C-PnC*. The *C-PnC* shows one complete acoustic bandgap in the frequency range from 67.7 to 83.5 MHz.

**Figure 4:**
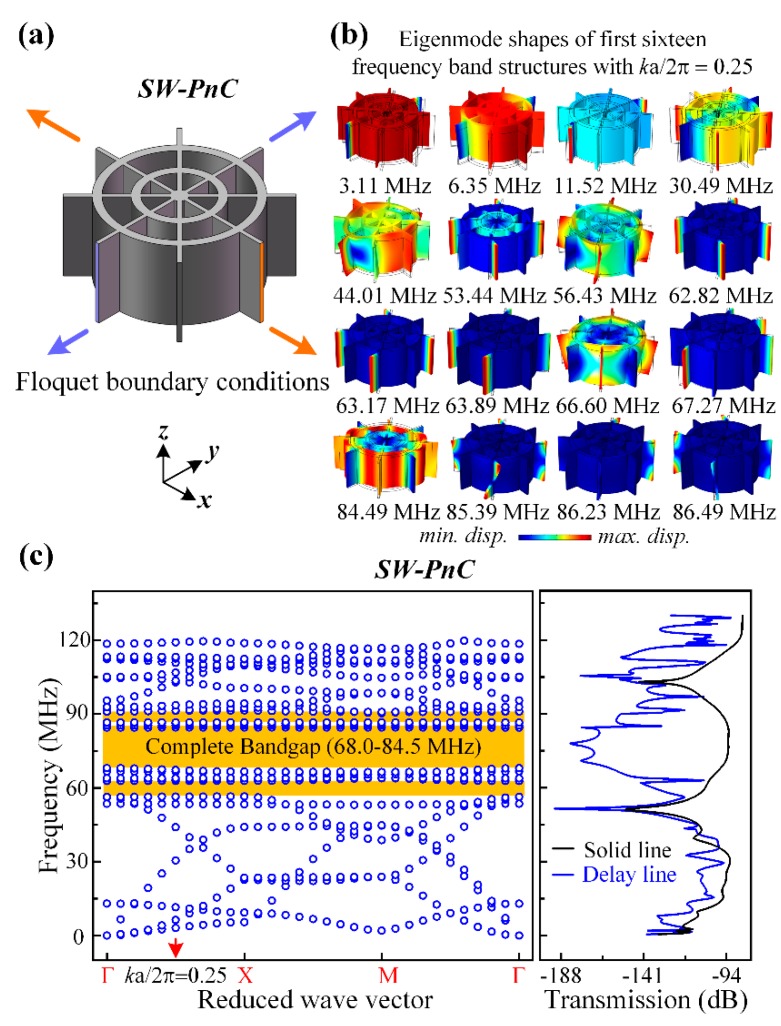
(**a**) 3D Illustration of the Floquet boundary conditions applied to a *SW-PnC* unit cell. Moreover, the boundary condition applied on the orange surfaces means an infinite number of repetitions in the *x*-direction, and the condition applied on the purple surfaces means an infinite number of repetitions in the *y*-direction. (**b**) Corresponding eigenmode shapes of the first 16 frequency band structures for the proposed *SW-PnC* with *k*a/2π = 0.25. (**c**) Band structures of an infinite PnC are consistent with the transmission characteristics through a finite number of PnC unit cells for *SW-PnC*. The *SW-PnC* shows three complete bandgaps, and the widest frequency range of them is from 68.0 to 84.5 MHz.

Figure 3 and Figure 4 present the band structures and transmission properties of the proposed PnCs. As shown in Figure 3a and Figure 4a, the Flouqet periodic boundary conditions were applied to a C-PnC unit cell and SW-PnC unit cell, respectively. Specifically, the boundary condition applied on the orange surfaces means an infinite number of repetitions in the *x*-direction, and the condition applied on the purple surfaces means an infinite number of repetitions in the *y*-direction. Moreover, the dispersion relations of C-PnC shows one complete acoustic bandgap in the frequency range from 67.7 to 83.5 MHz. The corresponding eigenmode shapes of the C-PnC of the first 10 frequency band structures with *k*a/2π = 0.25 are displayed in Figure 3b. Furthermore, as shown in Figure 4, the SW-PnC shows three complete bandgaps, and the widest frequency range of these is from 68.0 to 84.5 MHz. Subsequently, the corresponding eigenmode shapes of the SW-PnC of the first 16 frequency band structures with ka/2π = 0.25 are displayed in Figure 4b.

To investigate the peculiarity of the *SW-PnC* and verify that acoustic bandgaps can be formed by the proposed PnCs, transmission parameters (i.e., S21) of the delay line with four periods of the *SW-PnC* strip, the solid line with the solid strip (*SW-PnC*), delay line with three periods of the *C-PnC* strip, and the solid line with the solid strip (*C-PnC*) were performed. As shown in Figure 3c and Figure 4c, the FEA simulation result illustrates the wave attenuation mechanism in the transmission spectra, which is consistent with the associated acoustic bandgaps. It is noteworthy that the bandgap of PnC for out-of-plane modes is a partial bandgap for in-plane modes. For instance, as shown in Figure 3b,c, the last two frequency band structures of *C-PnC* are the out-of-plane mode, which means an in-plane bandgap can be achieved between the frequency of 104.23 MHz and the frequency of 123.83 MHz. Therefore, a wave attenuation mechanism can be observed at the frequency range round 120 MHz.

Herein, to measure the performance of associated PnC, the bandwidth BG% was introduced in this research and can be defined as the gap to mid-gap ratio [33]:

(3) $$\mathrm{BG}\%=\frac{\left(f_{\mathrm{up}}-f_{\mathrm{down}}\right)}{\left(\frac{f_{\mathrm{up}}+f_{\mathrm{down}}}{2}\right)}\%,$$

where *f_up_* and *f_down_* are the bounding frequencies of the acoustic bandgap. In this research, the frequency range of the complete bandgap for *C-PnC* is from 67.7 to 83.5 MHz and the frequency range of the largest complete bandgap for *SW-PnC* is from 68.0 to 84.5 MHz. Apparently, the mid-gap frequency of *C-PnC* is approximately equal as 76 MHz to that of *SW-PnC*. The BG% of *C-PnC* and *SW-PnC* is 20.9% and 21.6%, respectively. In general, the proposed *SW-PnC* possesses a smaller lattice constant (70.6%) and much lighter weight (44.2%) in a similar bandgap frequency range compared with *C-PnC*.

**Figure 5:**
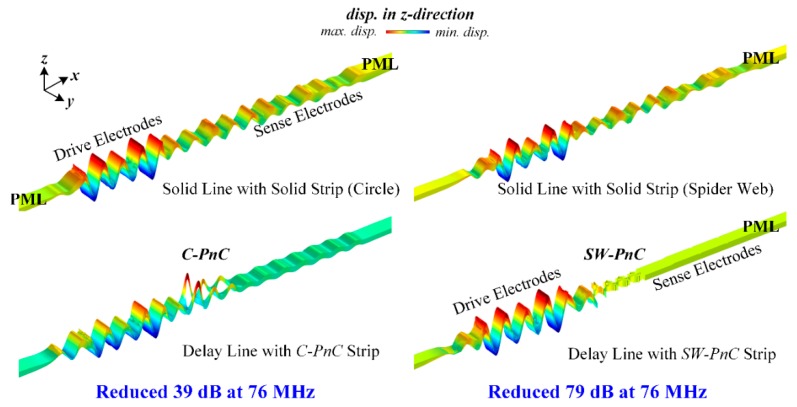
Illustration of the displacement distribution in the *z*-direction of two types of delay line and solid line working in the frequency of 76 MHz, and a more significant isolation of the acoustic wave in the delay line where a smaller-sized *SW-PnC* strip is observed. In general, due to the large circle hole, the *C-PnC* is already a very lightweight PnC structure compared with previously reported PnCs. Nevertheless, the proposed *SW-PnC* possesses a smaller lattice constant (70.6%), much lighter weight (44.2%), and greater energy loss suppression (two-fold) in a similar bandgap frequency range compared with *C-PnC*.

Figure 3c and Figure 4c show that the two PnC structures have a similar frequency range of acoustic bandgaps, but *SW-PnC* has a more significant isolation of acoustic waves. As illustrated in Figure 5, more insight into the wave isolation in the proposed PnCs is achieved by analyzing the displacement distribution in the *z*-direction of two types of delay line and solid line working at the frequency of 76 MHz. The left part of Figure 5 shows that the *C-PnC* strip can only suppress part of the acoustic wave propagation, indicating that it will still have a nonnegligible energy loss if applied in the MEMS resonator. As shown in the right of Figure 5, since the *SW-PnC* strip is applied in the delay line, the PnC transmission medium effectively prohibits the propagation of waves generated from the drive IDTs. In particular, the displacement amplitude in the *z*-direction in the domain of the sense electrodes is approximately zero, which means the *SW-PnC* strip can completely suppress the propagation of acoustic waves. Furthermore, through a numerical calculation of the simulation results, the delay line with the *SW-PnC* strip is reduced by 79 dB and the delay line with the *C-PnC* strip is only reduced by 39 dB at the same frequency of 76 MHz compared with that of the associated reference solid line, suggesting that a more significant (i.e., two-fold) reduction of energy loss was achieved by the *SW-PnC*.

**Figure 6:**
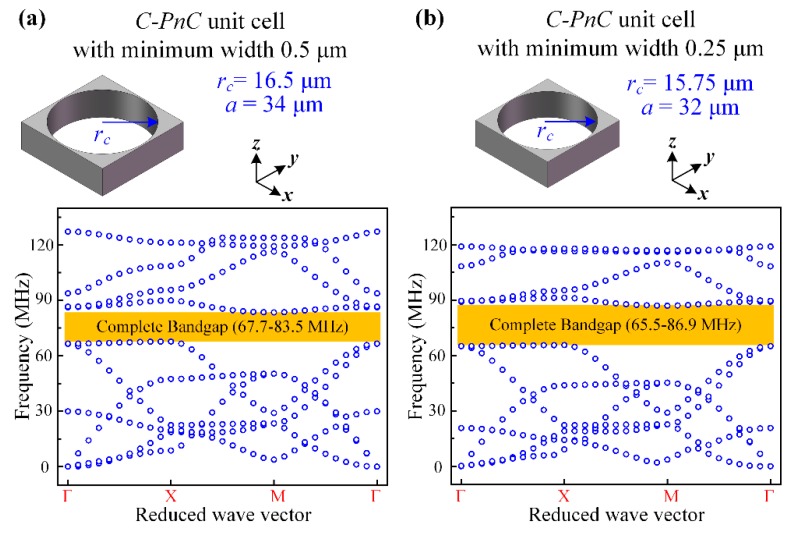
Dispersion relations of the *C-PnC* unit cell with a minimum width of (**a**) 0.5 μm and (**b**) 0.25 μm, respectively. Meanwhile, the mid gap frequency of these two kinds of *C-PnC* are approximately equal as 76 MHz.

In addition, it is worth noting that the minimum width in a single PnC cell (0.5 μm) is not the minimum width in the *C-PnC* array, which is a series of repeated cells. The minimum width of the *C-PnC* array is twice the minimum width of 0.5 μm. However, the minimum width of the *SW-PnC* array (0.5 μm) is the same as that of the *SW-PnC* unit cell. Given this, the acoustic bandgap of the *C-PnC* unit cell with a minimum width of 0.25 μm was calculated. As shown in Figure 6, to make the bandgap be in a similar frequency range, the lattice constant and radius of the circle hole of the *C-PnC* unit cell with a 0.25-μm minimum width was designed as 32 μm and 15.75 μm, respectively. Moreover, as shown in Figure 6b, the BG% of the *C-PnC* with a minimum width of 0.25 μm increased to 28.1%. Nevertheless, in comparison with the *C-PnC* (0.25 μm minimum width), the proposed *SW-PnC* still shows a smaller lattice constant (75%) and lighter weight (54.3%) with the same mid-gap frequency of 76 MHz. In this research, we were more focused on discussing the minimum width of the *SW-PnC* unit cell and *C-PnC* unit cell being the same as 0.5 μm. Thereby, the following discussions about the application of *C-PnC* in MEMS resonators is based on the 0.5-μm minimum width.

## 3. Resonators Design

**Figure 7:**
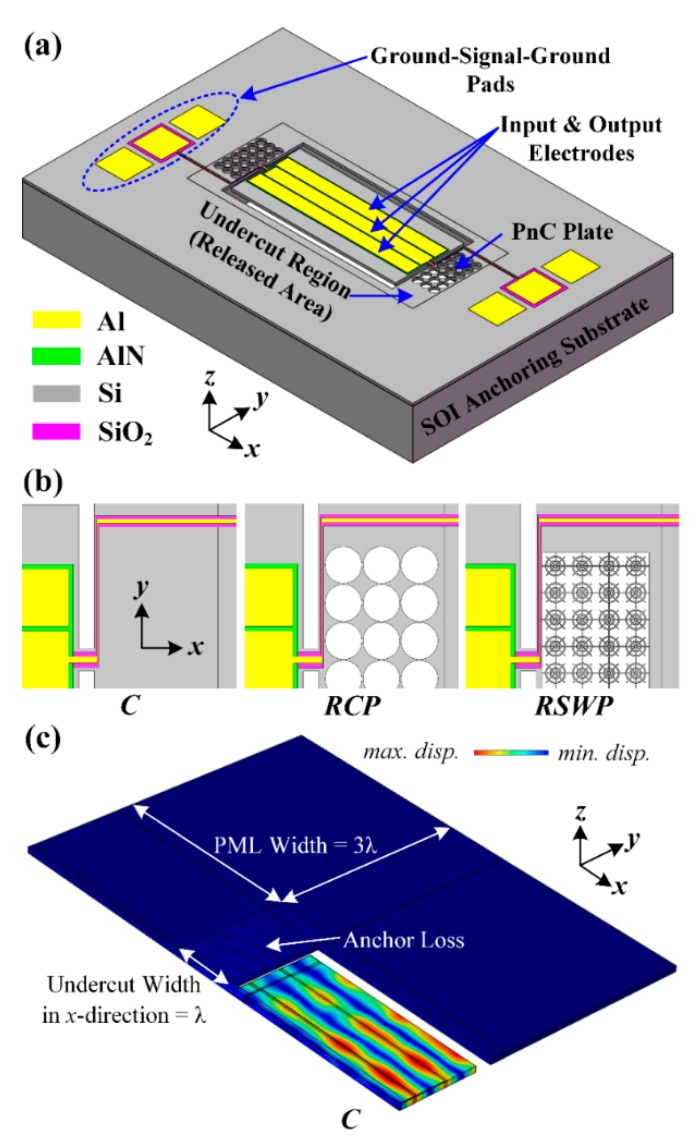
(**a**) 3D Illustration of the fifth-order symmetric lamb mode aluminum nitride (AlN)-on- silicon-on-insulator (SOI) MEMS resonator with *C-PnC* array plates in each of the undercut regions. (**b**) Three types of MEMS resonators, including the conventional resonator (C), the resonator with 3 × 8 *C-PnC* array plates (RCP), and with 4 × 10 *SW-PnC* array plates (RSWP) were designed in each of undercut regions to further verify the effectiveness of a finite PnC structure in reducing the anchor loss and indicating that the resonator RSWP could realize the optimal *Q*. In this research, (**c**) the width of perfectly matched layer (PML) and the width of the undercut region in the *x*-direction of resonators had a three-fold wavelength (3*λ*) and were identical to one *λ*, respectively. In addition, finite element analysis was only performed for a quarter section of the resonator due to the symmetric width-extensional resonant mode (a symmetric boundary condition was applied to the symmetric surfaces).

In order to further demonstrate the effectiveness of the proposed PnCs, as illustrated in Figure 7a,b, PnC array plates as the anchoring boundaries were designed in an AlN-on-SOI MEMS resonator to improve the quality factor (*Q*) by reducing the anchor loss compared with the conventional MEMS resonator. In this research, the center-to-center electrode pitch (W_p_) of resonators was set as 56 µm, and designed to be transduced at the fifth-order symmetric lamb mode, which resonates at the frequency of 76 MHz. The resonant frequency of resonators for any given harmonic mode is given by [34]:

(4) $$f=\frac{nv}{2W_{r}},$$

where *W*_r_ is the width of the resonator and *n* is the number of harmonic modes. For a fifth-order symmetric mode of resonators, the *W*_r_ is equal to the five-fold W_p_ (i.e., W_r_ = 5 W_p_).

Moreover, three types of MEMS resonators with the same size of undercut regions, including the conventional resonator (C), the resonator with 3 × 8 *C-PnC* array plates (RCP), and the resonator with 4 × 10 *SW-PnC* array plates (RSWP), were designed in each of the undercut regions to further verify that the resonator that applied the *SW-PnC* could realize the optimal *Q*. In this research, all the simulation models of resonators were obtained according to the PMLs boundary conditions to absorb the dissipated acoustic waves, and the anchor loss (Q_anc_) of resonators was calculated by [35]:

(5) Qanc=Re(ω)2Im(ω),

where *ω* is the eigenfrequency of the desired resonant mode of resonators. In addition, as shown in Figure 4c, FEA simulation was only performed for a quarter section of the resonator due to the symmetric width-extensional (WE) resonant mode (i.e., a symmetric boundary condition was applied to the symmetric surfaces).

In addition, the lightness of the PnC structure in MEMS resonators seems to be an unimportant merit in this research. However, as mentioned in [18], it was found that the PnC arrays in the suspended frame structure or on the supporting tethers are more effective in reducing the anchor loss compared with conventional 1D PnC-based tethers and utilizing 2D PnC plates as anchoring substrates. Given this, the lightweight PnC indicates a potential application in MEMS resonators to improve structural stability, and to avoid undesired spurious modes introduced by the PnC.

## 4. Results and Discussion

**Figure 8:**
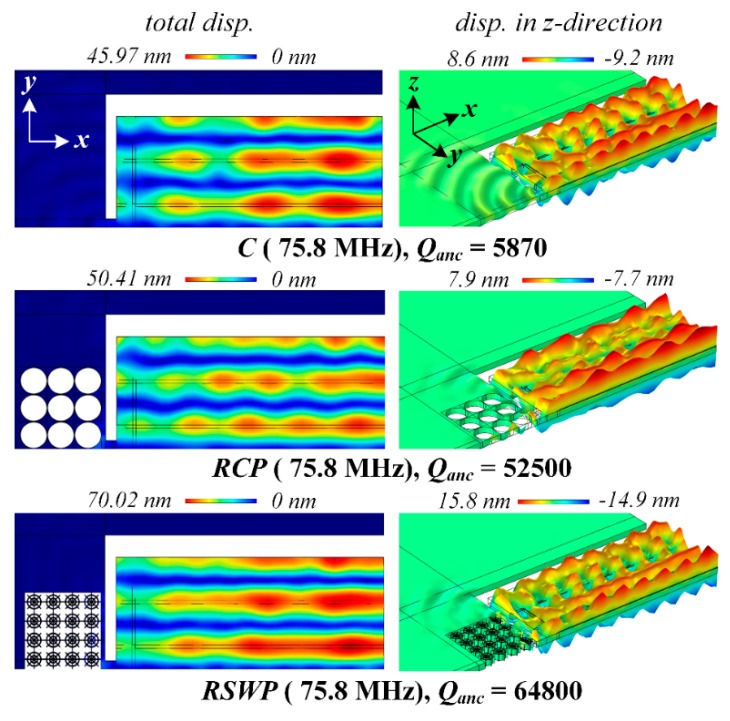
Illustration of displacement distributions of the fifth-order width-extensional resonant mode of the conventional resonator (C), resonator with *C-PnC* array plates (RCP), and resonator with *SW-PnC* array plates (RSWP). It is noteworthy that the left side and right side are total displacement and displacement in the *z*-direction distributions, respectively. The simulated anchor loss (*Q_anc_*) of resonators agrees well with the transmission property of the delay line and solid line.

Figure 8 shows the displacement distributions of the resonant mode of resonator C, RCP, and RSWP. The design C shows the lowest *Q*_anc_ of 5870, indicating a large part of the acoustic wave dissipated through supporting tethers. The resonator RCP shows a relatively higher *Q*_anc_ of 52,500, and the device RSWP with the *SW-PnC* array plate shows the most significant displacement isolation in undercut regions, as well as the anchoring substrate, with a *Q*_anc_ of up to 64,800. From the above discussion, the simulated *Q*_anc_ of resonators agrees well with the transmission properties of the delay line and solid line.

Furthermore, simulated admittance Y11 curves of resonators were calculated by the FEA frequency domain simulation under a 50-Ω load to extract the series resonant frequency (*f*_s_), loaded *Q*, motional resistance (*R*_m_), and the effective electromechanical coupling coefficient (*k^2^*_eff_). The relationships among the *f*_s_, *Q*, *R*_m_, and *k^2^*_eff_ were defined as [36,37,38]:

(6) Q=fsΔf-3dB, Rm=1max{Re(Y11)}, keff2=fp2−fs2fp2,

where ∆*f_-3dB_* is the −3dB bandwidth around the series resonant frequency. The max {Re(Y11)} and *f_p_* refer to the maximum value of the real part for the admittance Y11 and the parallel resonant frequency of resonators, respectively.

**Figure 9:**
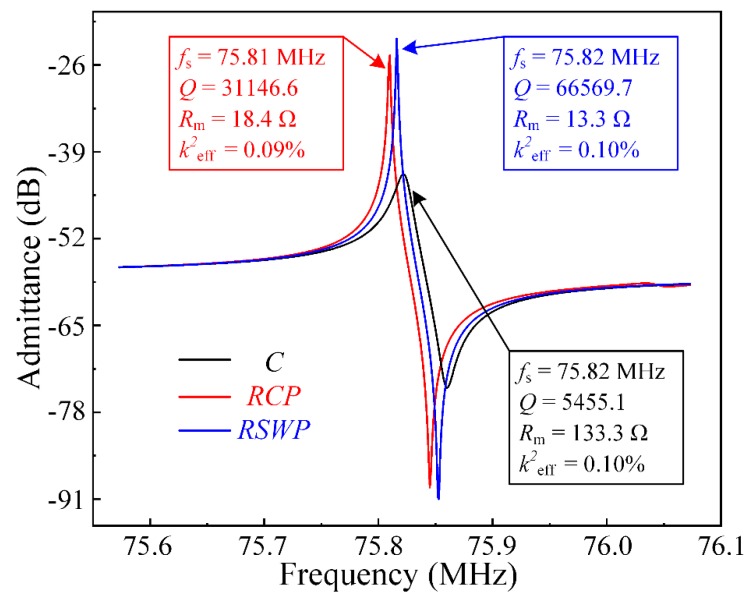
Illustration of the admittance spectra for resonators C, RCP, and RSWP in the same frequency range. The simulated results of admittance curves are well consistent with the transmission characteristics, verifying that the SW-PnC can sufficiently reduce the energy dissipation of resonators, thereby achieving the optimal *Q*.

As shown in Figure 9, the *Q* of resonator RCP of 31,146.6 was 5.7 times larger than that of resonator C, which means most of the dissipated acoustic waves were reflected to the resonant body. It is worth noting that the *Q* only considered the anchor loss (i.e., ignored thermo-elastic dissipation, viscous loss, etc.). Moreover, the *Q* of the resonator RSWP with the *SW-PnC* array plate was 66,569.7 at 75.82 MHz and showed an improvement of 12.2 times compared with that of C, while the motional resistance decreased to 13.3 Ω. In brief, the systematical investigation by the FEA simulation revealed that the application of *SW-PnC* effectively reduced the anchor loss, and realized a satisfactory *Q*.

## 5. Conclusions

In summary, a 2D spider web-like PnC structure was proposed in this research. The band structures and transmission characteristics of the PnCs were systematically analyzed by using FEA simulations. In comparison with the PnC with circle holes in a similar frequency range of the acoustic bandgap, the proposed PnC induced a much smaller lattice constant (70.6%), much lighter weight (44.2%), and greater reduction of energy dissipation (two-fold). At the same time, three types of MEMS resonators were designed to further verify that the resonator with the SW-PnC array plate as the anchoring boundaries realized the optimal *Q* as high as 66,569.7 at 75.82 MHz, indicating a 12.2-fold improvement compared with the conventional one. These results reveal the attractive potential application of miniaturization in MEMS devices by the proposed SW-PnC.
